# A retrospective cross-sectional study of type 2 diabetes overtreatment in patients admitted to the geriatric ward

**DOI:** 10.1186/s12877-019-1256-2

**Published:** 2019-09-02

**Authors:** Zyta Beata Wojszel, Agnieszka Kasiukiewicz

**Affiliations:** 1 Department of Geriatrics, Medical University of Bialystok, Fabryczna str. 27; 15-471, Bialystok, Poland; 2 Department of Geriatrics, Hospital of the Ministry of Interior in Bialystok, Bialystok, Poland

**Keywords:** Type 2 diabetes mellitus, Overtreatment, Older people, Hypoglycemia risk factors, Glycated hemoglobin A1c

## Abstract

**Background:**

Glycemic control targets in older patients should be individualized according to functional status and comorbidities. The aim of the study was to identify high-risk patients who had evidence of tight glycemic control and thus at risk of serious hypoglycemia.

**Methods:**

Retrospective cross-sectional study of type 2 diabetes patients admitted to the geriatric ward receiving diabetes medications. Patients’ hospital records were analyzed. The high risk of hypoglycemia group constituted patients who were aged 80+ years, diagnosed with dementia, with end- stage renal disease, or with a history of macrovascular complications. The primary outcome measure was hemoglobin A_1C_ (HbA_1C_) ≤ 7.0% [53 mmol/mol].

**Results:**

Two hundred thirteen patients were included (77.5% women; 49.3% 80+ year-old). 65.3% received sulfonylurea, 39,4%- metformin, 32.9%- insulin, and 4.2%- acarbose (in 61.5% as monotherapy, and in 38.5% combination therapy). We identified 130 patients (60%) as the denominator for the primary outcome measure; 73.1% had a HbA_1C_ value ≤7.0% [53.3 mmol/mol], but 55.4% ≤6,5% [48.8 mmol/mol], and 40.8% ≤6.0% [42 mmol/mol].

**Conclusions:**

The results show a very high rate of tight glycemic control in older patients admitted to the geriatric ward, for whom higher HbA_1C_ targets are recommended. This indicates the high probability of diabetes overtreatment in this group, associated with a high risk of recurrent hypoglycemia. This is all the more likely because most of them received medications known to cause hypoglycemia. This points to the need of paying more attention to specific difficulties in diabetes treatment in older people, especially those suffering from various geriatric syndromes and diseases worsening their prognosis.

## Background

Age is one of the main risk factors for type 2 diabetes mellitus. The prevalence of diabetes is the highest (10–20%- depending on the population and methods used in the study) in people over 70 years old [1]. In the Multicenter Polish Population Heath Status Study, which involved a population of adult Poles aged 20–74 years, the overall prevalence of diabetes was 6.8%, whereas it was 16.3% (in males) and 17.8% (in females) for individuals over 60 years old [2]. In the Polish national PolSenior study, conducted among 4979 participants aged 65 and over, the prevalence of type 2 diabetes for 65–79 year old people and 80 and older was 18.7 and 17% respectively [3].

Diabetes and its complications, mainly cardiovascular diseases, are among the five leading causes of death in developed countries [4]. Thus, for many years the efforts of researchers and diabetologists have been focused on both prevention of development of type 2 diabetes and glycemic control, which could prevent diabetic vascular complications. The necessity of intensive type 2 diabetes treatment was suggested for the first time following the reports on the results of the UKPDS study, which demonstrated beneficial effects of such actions on microvascular complications [5]. However, the expected benefits in relation to cardiovascular diseases were not supported by three subsequent large prospective studies (ACCORD [6], ADVANCE [7], and VADT [8]) published in 2008–2009. A meta-analysis of these studies showed that mainly younger patients with recently diagnosed diabetes and no previous macrovascular complications experienced the benefits of intensive type 2 diabetes treatment. Instead of a reduced risk of cardiovascular diseases, a growing risk of severe hypoglycemia was reported in patients with a number of coexisting diseases and long-term diabetes [9]. This supports the need for early diagnosis followed by intensive therapy of diabetes, but also indicates that the benefits of such treatment are limited in the older population of patients with a long-term disease and short expected survival [10, 11]. Therefore in recent years, there have been discussions among diabetes associations with regard to the target levels of glycemic control, especially in the older population. The necessity of therapy individualization based on patients’ characteristics was emphasized, in particular- their ability to identify and manage hypoglycemia. In older patients, this ability may be significantly adversely affected by common geriatric comorbidities such as functional disability, depression or dementia, to which diabetes predisposes [12, 13]. These comorbidities also adversely affect long-term survival prognosis [14]. In 2011 year the European Diabetes Working Party for Older People (EDWPOP) based the therapeutic goals in diabetes on the older patient’s status, and recommended HbA_1C_ levels of 7–7.5% [53-58 mmol/mol] for older patients in good health, and 7.6–8.5% [60-69 mmol/mol] for the frail ones [15].

The aim of the study was to assess the prevalence of tight glycemic control in older patients taking diabetes medications on admission to the ward, and to identify “high-risk patients” who had evidence of tight glycemic treatment and thus were at risk of serious hypoglycemia. The study covered the years 2009–2010, when- according to the guidelines of the Diabetes Poland Association- HbA_1C_ ≤ 7% [53 mmol/mol] was the general recommended therapeutic goal for type 2 diabetes. The lower HbA_1C_ value, i.e. < 6.5% [48 mmol/mol], was suggested only in patients with type 1 and newly diagnosed type 2 diabetes, or with a short history of the disease [16, 17]. It was only in 2011 that the Diabetes Poland Association in its diabetes treatment guidelines established the third diabetes treatment goal, i.e. HbA_1C_ < 8% [64 mmol/mol] in older patients with long-term diabetes and vascular complications [18]. However, it should be emphasized that, although the Polish guidelines for diabetes treatment previously in force and recommended by the national consultants in the field of family medicine and diabetology did not contain separate therapeutic targets for older patients, they pointed out that in this population- especially in case of significant comorbidities and if their survival prognosis is less than 10 years- it may be necessary to lower the glycemic control criteria to a level that does not lead to worsening of the patients’ quality of life [19]. For the above reasons, it could be expected that the group of people diagnosed with HbA_1C_ equal or lower than 7% [53 mmol/mol] would be characterized by significantly better parameters of health and psycho-physical abilities than the group of people with HbA_1C_ levels higher than 7% [53 mmol/l].

To the authors’ knowledge, this is the first publication focused on the assessment of the implementation of the principle of diabetes therapy individualization in late old age in everyday practice in Poland.

## Methods

### Setting, inclusion criteria

We retrospectively analyzed medical records of all consecutive patients admitted to the geriatric ward of the Hospital of the Ministry of Interior in Bialystok, Poland, between 1st January, 2009 and 31st December 2010, and discharged with the diagnosis of type 2 diabetes according to ICD10. The study population included patients on insulin and/or oral glucose lowering drugs before admission, who had hemoglobin A_1c_ value documented in the medical record. HbA1c measurements were made with the immunoinhibition method using an Olympus AU400 analyzer.

The department of geriatrics is a sub-acute care ward. Older people with multimorbidity and accompanying physical disability and/or cognitive impairment are referred to it, and are admitted mainly in a planned manner (the average time from referral until admission is 3 months). The mean length of stay at the department is 7 days. A comprehensive geriatric assessment by the multidisciplinary team, including a review and modification of patient’s pharmacotherapy, is performed during the patients’ hospitalization.

### Study parameters

We identified a “high risk of hypoglycemia group”- patients with diabetes treated with antidiabetic medications and having at least one of the following additional criteria: age 80 years or older, severe stage of chronic kidney disease (CKD) i.e. stage 4 and 5 CKD according to Kidney Disease Outcome Quality Initiative (KDOQI)- glomerular filtration rate GFR < 30 ml/min/1.73m^2^, a diagnosis of dementia confirmed at discharge, a history of cardiovascular or vascular complications (myocardial infarction, coronary artery bypass graft- CABG, percutaneous transluminal coronary angioplasty- PTCA, stroke, transient ischemic attack- TIA). People who did not meet these criteria were classified as “low risk group”. We defined tight treatment (primary outcome measure) in these patients based on their HbA_1C_ value at admission, using 3 different thresholds (≤7.0% [53 mmol/mol] and > 6.5% [48 mmol/mol]; ≤6.5% [48 mmol/mol] and > 6.0% [42 mmol/mol]; and ≤ 6.0% [42 mmol/mol]), which reflect increasingly tight glycemic control and are associated with increasing risk of hypoglycemia in older diabetic patients [20].

Data on patients’ age, gender, place of residence (urban/rural), physical and psychological abilities (based on comprehensive geriatric assessment scales: Barthel Index [21], 6-point instrumental activities of daily living scale (IADL) derived from Duke OARS scale [22], Norton scale [23], Short Orientation- Memory- Concentration Test- SOMCT [24] and Geriatric Depression Scale- GDS [25]), and parameters of nutritional status (body mass index- BMI, number of lymphocytes in blood), renal function (GFR counted using the Cockroft-Gault formula [26], serum creatinine level), and HbA_1c_ level were collected. The prevalence of dementia (confirmed in neuropsychological examination), depression, hypertension and orthostatic hypotension as well as serious macrovascular complications (such as myocardial infarction, CABG or PTCA, stroke or TIA) was evaluated based on diagnoses and information in the discharge summaries. Hypoglycemic agents use (insulin, sulfonylurea, metformin, and others), and medication regimen (monotherapy; combination therapy, and specification of combinations used) both prior to admittance and recommended at discharge were evaluated.

### Statistical analysis

Data were collected and analyzed using IBM SPSS Version 18 Software suit (SPSS, Chicago, IL, USA), and presented as means and standard deviation for normally distributed, as medians and interquartile range for not normally distributed continuous variables, and the number of cases and percentage for categorical variables. Proportions were compared using χ2 tests, while the independent samples t-test and Mann-Whitney U test were used to compare measures of central tendency (means and medians). A multivariable logistic regression including all predictors of HbA1_c_ ≤ 7% [53 mmol/mol] with a *P* value less than 0.2 was performed. To assess differences between two dependent variables, Wilcoxon signed-rank test was used. A *P* value of less than 0.05 was regarded as significant.

## Results

**Fig. 1:**
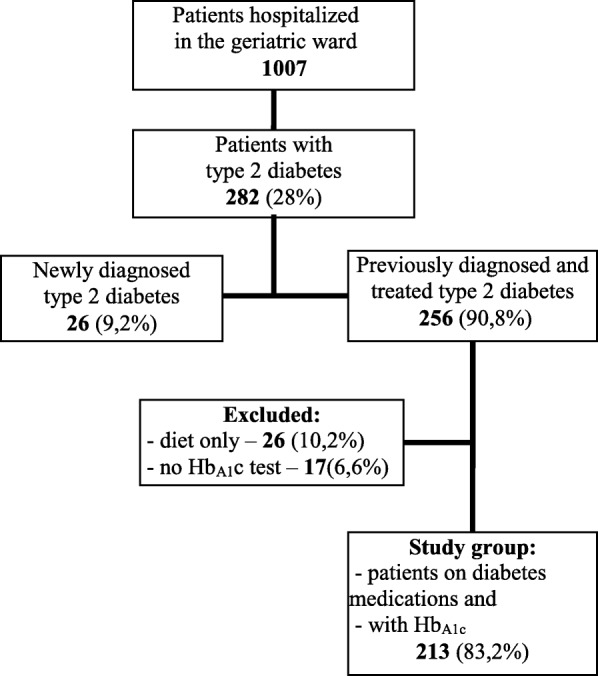
Flow chart of patients enrollment

Two hundred thirteen patients who were treated with insulin and/or oral glucose lowering agents before hospitalization, and who had HbA_1c_ test result performed at admission, were included in the study- Fig. 1 shows patients enrolment in the study. Most were women (165; 77.5%), and almost a half of them were aged 80 years or more (105; 49.3%). The mean age was 78.9 (±6.5) years. All patients were community dwelling and people living in rural area constituted only 11.3% of the group.

**Fig. 2:**
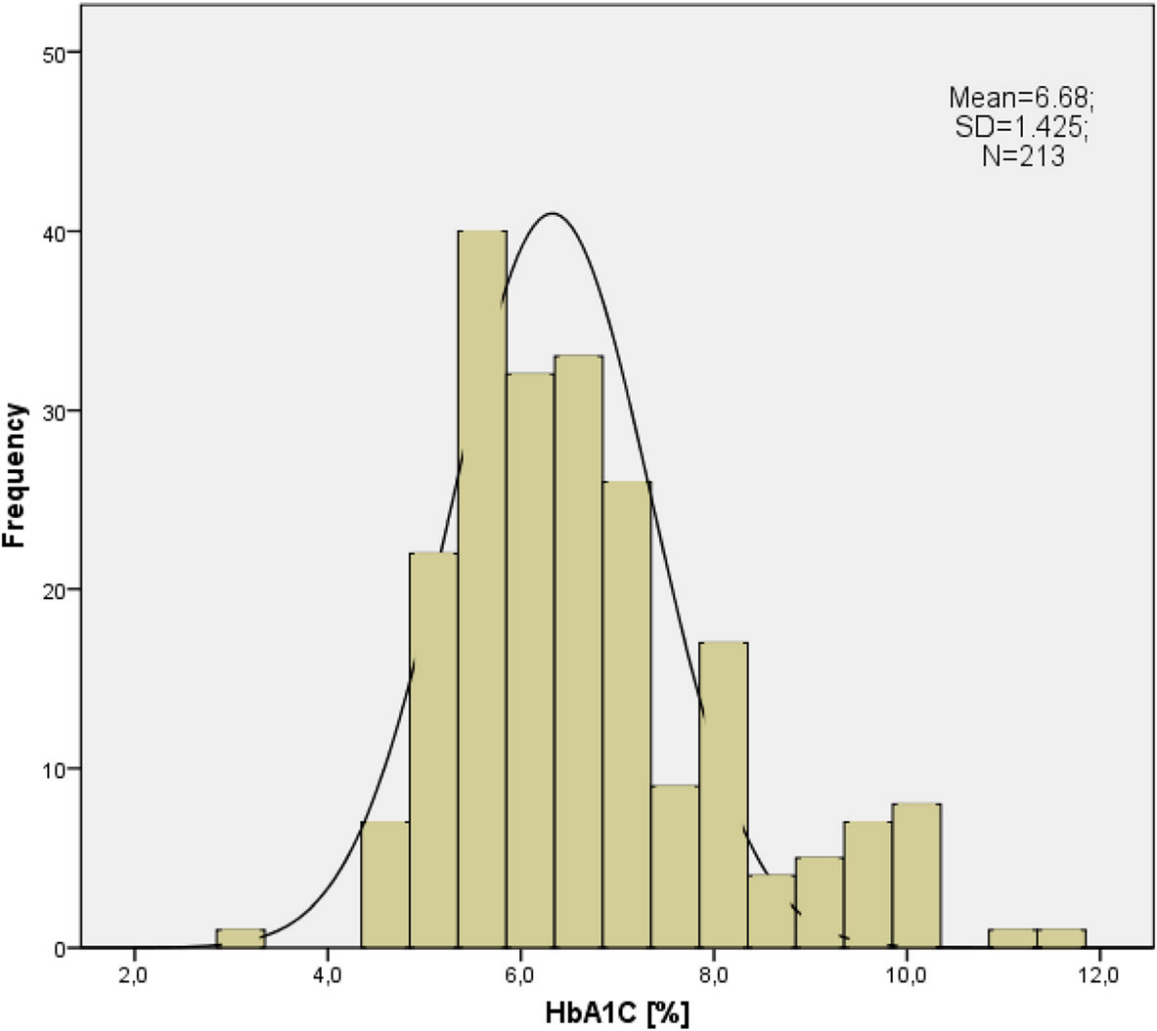
The distribution of HbA1_c_ values in the study group

The median value of HbA_1C_ level was 6.4% [46 mmol/mol], minimum 3.1% [10 mmol/mol], maximum 11.6% [103 mmol/mol], interquartile range 5.6–7.3% [38–56 mmol/mol]. The lowest HbA1 C (one case) was observed in patient on gliclazide, suffering from hemolytic anemia, periodically on steroids (without this treatment prior to hospitalization), with liver failure, and suspicion of secondary adrenal insufficiency. A number of elements could actually cause severe hypoglycemia and low HbA1c in this patient, but also in some way affect HbA1c measurement. A relatively small part of patients admitted to the geriatric ward (34 patients;16%) had HbA_1C_ above 8% [64 mmol/mol]. In the majority of cases (148; 69.5%) HbA_1C_ level was ≤7% [53 mmol/mol] (Fig. 2). To assess the homogeneity of the groups and evaluate the possible risk factors connected with the most intensive treatment of type 2 diabetes, we compared patients with HbA_1C_ ≤ 7% [53 mmol/mol] with patients with HbA_1C_ >  7% [53 mmol/mol] (Table 1).

**Table 1:** Characteristics of older patients admitted to the geriatric ward by level of glycemic control

Patient characteristics	All patients	Tightly controlled; HbA_1C_ ≤ 7% [53 mmol/mol]	Not tightly controlled; HbA_1C_ > 7% [53 mmol/mol]	*P* value^a^
No. (%) of patients	213 (100.0)	148 (69.5)	65 (30.5)	
Age, years, M(SD)	78.9(6.5)	79.3 (6.3)	78.0 (6.9)	0.19
Age, 80 + years	105 (49.3)	77 (52.0)	28 (43.1)	0.24
Female sex	165 (77.5)	111 (75.0)	54 (83.1)	0.22
Place of residence, rural	24 (11.3)	14 (9.5)	10 (15.4)	0.24
Barthel Index score	90 (70–100)	90 (70–100)	90 (80–100)	0.82
IADL score	8 (5–11)	8 (5–11)	8 (6–11)	0.52
Norton scale score	17 (15–19)	17 (15–19)	17 (16–19)	0.60
Pressure sores risk	36 (17.2)	28 (19.3)	8 (12.5)	0.32
SOMCT score	6 (1–14)	6 (1–14)	8 (2–12)	0.98
Dementia	40 (18.8)	32 (21.6)	8 (12.3)	0.13
GDS score, M(SD)	6.98 (3.6)	6.78 (3.5)	7.43 (3.8)	0.23
Depression	138 (64.8)	99 (66.9)	39 (60.0)	0.35
Hypertension	190 (89.2)	130 (87.8)	60 (92.3)	0.47
Orthostatic hypotension	28 (15.6)	17 (13.1)	11 (22.4)	0.17
Macrovascular complications	44 (20.7)	31 (20.9)	13 (20.0)	0.88
MI, CABG, PTCA	19 (8.9)	14 (9.5)	5 (7.7)	0.80
TIA	19 (8.9)	13 (8.8)	6 (9.2)	0.92
Stroke	25 (11.7)	16 (10.8)	9 (13.8)	0.64
BMI, kg/m^2^	32 (28.0–36.5)	31 (27.3–35.8)	33 (30.0–39.8)	0.11
Lymphocytes, K/μL, M(SD)	1.87 (0.73)	1.76 (0.70)	2.13 (0.74)	0.001
Lymphocytes < 1.5 K/μL	64 (30.8)	52 (36.1)	12 (18.8)	0.01
GFR^b^, ml/min/1.73m^2^, M(SD)	55 (20.97)	54.18 (20.94)	56.82 (21.10)	0.42
GFR categories, ml/min/1.73m^2^				
eGFR < 30	23 (12.0)	19 (14.4)	4 (6.7)	0.15
GFR < 60	118 (61.5)	81 (61.4)	37 (61.7)	0.97
Serum creatinine, μmol/L	95.5 (78.7–114.0)	93,7 (77.8–111.4)	101.7 (81.3–120.2)	0.1
HbA_1C_				
HbA_1C_[%]	6.4 (5.6–7.3)	5.9 (5.4–6.5)	8.1 (7.5–9.4)	< 0.001
HbA_1C_ [mmol/mol]	46 (38–56)	41 (36–48)	65 (58–79)	
Medications class at admittance				
Insulin	70 (32.9)	33 (22.4)	37 (56.9)	< 0.001
Metformin	84 (39.4)	62 (41.9)	22 (33.8)	0.29
Sulfonylurea	139 (65.3)	102 (68.9)	37 (56.9)	0.12
α-Glucosidase inhibitors	9 (4.2)	6 (4.1)	3 (4.6)	0.85
Medications class at discharge				
Insulin	71 (33.5)	30 (20.4)	41 (63.1)	< 0.001
Metformin	110 (51.9)	66 (44.9)	44 (67.7)	0.12
Sulfonylurea	122 (57.5)	86 (58.5)	36 (55.4)	0.76
α-Glucosidase inhibitors	3 (1.4)	3 (2.0)	–	0.55

N (%) or median values (IQR) are shown unless otherwise indicated; ^a^χ^2^ test or Fisher exact test, as appropriate, for categorical variables, and Mann-Whitney test or t-test for continuous variables, as appropriate; ^b^Cockroft-Gault equation was used to calculate the GFR; *BMI* body mass index, *CABG* coronary artery bypass graft, *GDS* Geriatric Depression Scale, *GFR* glomerular filtration rate, *HbA*_*1C*_ glycosylated A_1C_ hemoglobin, *IADL* instrumental activities of daily living, *IQR* interquartile range, *M* mean, *MI* myocardial infarction, *N* number of cases, *PTCA* percutaneous transluminal coronary angioplasty, *SD* standard deviation, *SOMCT* Short Orientation Memory Concentration Test, *TIA* transient ischemic attack

The groups did not differ in age, gender, place of residence, psycho-physical ability parameters (Barthel Index, instrumental activities of daily living scale, Norton scale, SOMCT and GDS scores), prevalence of hypertension, orthostatic hypotension, macrovascular complications, dementia or depression. The lymphocytes number was significantly lower and the prevalence of patients with lymphocytes below 1.5 K/μL was significantly higher in the group with the lower HbA_1C_ levels. The groups did not differ in the frequency of sulfonylurea, metformin and acarbose usage, but in the group with the higher values of HbA_1C_ insulin was used significantly more frequently and in multivariable logistic regression analysis, including all predictors of HbA1_c_ ≤ 7% [53 mmol/mol] with a *P* value less than 0.2, a significant independent negative effect associated with the lower values of HbA1_c_ was observed among patients on insulin (odds ratio, 0.32; 95% CI, 0.12–0.84; *P* = 0.02), when controlling for age, prevalence of dementia, orthostatic hypotension, lymphocytes < 1.5 K/μL, eGFR < 30 ml/min/1.73m^2^, BMI, and taking sulfonylureas (Table 2).

**Table 2:** Risk factors associated with low HbA_1C_ level (≤7% [53 mmol/mol])- multivariable logistic regression model

	OR	95% CI	*P* value
Age, years	1.0	0.94–1.07	0.99
Dementia	2.45	0.62–9.64	0.20
Orthostatic hypotension	0.53	0.19–1.47	0.22
BMI, kg/m^2^	0.99	0.97–1.02	0.59
Lymphocytes < 1.5 K/μL	1.99	0.81–4.93	0.14
GFR < 30 ml/min/1.73m^2^	2.63	0.59–11.8	0.21
Sulfonylurea	1.38	0.52–3.64	0.52
Insulin	0.32	0.12–0.84	0.02

*BMI* body mass index, *CI* confidence interval, *GFR* glomerular filtration rate, *OR* odds ratio

We identified 130 patients (61.03% of the total group) as the denominator for the primary outcome measure (age 80 years or older, or severe stage of chronic kidney disease, a diagnosis of dementia confirmed at discharge, a history of severe cardiovascular or vascular complications); 40.8% of them had HbA_1C_ value ≤6.0% [42 mmol/mol], 55.4% - ≤6,5% [48 mmol/mol], and 73.1% - ≤ 7.0% [53 mmol/mol]. The median level of HbA_1C_ in this group was 6.4% [46 mmol/mol], interquartile range 5.7–7.3% [39–56 mmol/mol] (Table 3). The ““high risk group” and the “low risk group” did not differ in the percentage of tight treatment prevalence and in median value of HbA_1C_. The percentage of patients with HbA_1C_ values within the range >  7% [53 mmol/mol] and ≤ 8% [64 mmol/mol] in the “high risk” group was only 11.5% and in the “low risk” group-19.3%, and only in small percentage of cases HbA_1C_ levels were higher than 8% (64 mmol/mol)- in 15.4 and 16.8% of cases respectively.

**Table 3:** HbA_1C_ categories by risk of hypoglycemia groups

HbA_1C_ category	All patients (*n* = 213)	High risk group^a^ (*n* = 130)	Low risk group^b^ (*n* = 83)	*P* value^c^
≤ 6.0% [42 ≤ mmol/mol]	40.8	40.8	41.0	0.39
> 6.0% [42 ≤ mmol/mol] and ≤ 6.5% [48 ≤ mmol/mol]	13.1	14.6	10.8	
> 6.5 [48 ≤ mmol/mol] and ≤ 7.0%, [53 ≤ mmol/mol]	15.5	17.7	12.0	
> 7.0%, [53 ≤ mmol/mol]	30.5	26.9	36.1	
HbA_1C_ [%]	6.4 (5.6–7.3)	6.4 (5.7–7.3)	6.5 (5.5–7.5)	0.84
HbA_1C_ [mmol/mol]	46 (38–56)	46 (39–56)	48 (37–58)	

N (%) or median values (IQR) are shown; ^a^High risk group- aged 80+ years or demented or with GFR < 30 ml/min./1.73 m2 or with macrovascular complications (stroke, TIA, PTCA, CABG, myocardial infarction); ^b^Low risk group- aged< 80 years, without dementia, without macrovascular complications and with GFR ≥ 30 ml/min./1.73 m2; ^c^χ^2^ test or Mann-Whitney test, *IQR* interquartile range, *N* number of cases

Before admitting to the ward the majority of patients received sulfonylurea (65.3%), less frequently metformin (39.4%) and insulin (32.9%) (Table 4). Acarbose was used only by 4.2% of them, and other therapeutic options were absent. At discharge metformin was used significantly more frequently (in 33–15% of cases- the drug was started), whereas sulfonylurea and acarbose were used significantly less frequently- discontinued respectively in 24 (11.3%) and in 7 (3.3%) cases. In 61.5% of subjects diabetes was treated with monotherapy before hospitalization, most frequently – with sulfonylurea (32.4% of cases). Combination therapy (with the most common combinations of sulfonylurea with metformin, sulfonylurea with insulin, and metformin with insulin) was used in 38.5% of patients. At discharge in 15 (6.6%) patients diabetes medications were stopped and only diabetic diet was recommended. A smaller percentage of subjects remained also on monotherapy.

**Table 4:** Glucose lowering medications use and medication regimen at admittance and at discharge from the geriatric ward

Medication	At admittance	At discharge	*P*^a^ value	Drug started	Drug discontinued
Insulin	70 (32.9)	71 (33.3)	0.74	4 (1.9)	5 (2.4)
Metformin	84 (39.4)	110 (51.6)	< 0.001	33 (15.6)	6 (2.8)
Sulfonylurea	139 (65.3)	122 (57.3)	0.01	8 (3.8)	24 (11.3)
α-Glucosidase inhibitors	9 (4.2)	3 (1.4)	0.03	1 (0.5)	7 (3.3)
Medication regimen					
Diet alone	–	15 (7.0)			
Monotherapy	131 (61.5)	106 (49.8)			
Sulfonylurea (SU)	69 (32.4)	53 (24.9)			
Insulin (I)	36 (16.9)	27 (12.7)			
Metformin (M)	24 (11.3)	25 (11.7)			
α-Glucosidase inhibitors (A)	2 (0.9)	1 (0.5)			
Combination therapy	82 (38.5)	92 (43.2)			
SU + M	44 (20.7)	47 (22.1)			
SU + I	17 (8.0)	6 (2.8)			
M + I	11 (5.2)	22 (10.3)			
SU + M + I	3 (1.4)	15 (7.0)			
SU + M + A	2 (0.9)	1 (0.5)			
I + A	1 (0.5)	1 (0.5)			
SU + A	2 (0.9)	–			
SU + A + I	2 (0.9)	–			

Data are N (%); ^a^Wilcoxon signed-rank test

## Discussion

Our study in patients admitted to the geriatric ward confirmed that “too tight” diabetes control was a far more common problem in this group than “unsatisfactory” control. The HbA_1C_ ≤ 7% [53 mmol/mol] was observed in almost 70% of the study participants on diabetes medications. Our study results are in line with the results of studies carried out in the USA and covering patients with type 2 diabetes in the age of 65 years or older with comorbid dementia, which covered the years 2008–2009, which found that one half had HbA_1C_ < 7% [53 mmol/mol] despite clear guidelines recommending higher glycemic targets, and the majority of the used medications that further exacerbated their potential risk for hypoglycemia [27], and with the results of NHANES study [28]. The HbA_1C_ ≤ 6% [42 mmol/mol] was observed in 40% of our study participants on glucose lowering agents, which, as shown in DCCT [29] or UKPDS [5] studies, is generally associated with increased risk of hypoglycemic episodes. HbA_1C_ > 8% [64 mmol/mol] (suggesting unsatisfactory diabetes control) was noted only in 16% of cases.

Contrary to our expectations, our research did not confirm the fact that diabetes therapy was indeed individualized in the case of the frail people in late old age. The group of people with HbA_1C_ ≤ 7% [53 mmol/mol] did not differ significantly in terms of general socio-demographic characteristics, health status and psycho-physical ability from people with higher HbA_1C_ values. The mean age, Barthel Index score, instrumental activities of daily living scale score, the prevalence of dementia, severe renal dysfunction or macroangiopathic complications was almost the same in both groups. Only lymphocytes number lower than 1.5 K/μl, suggesting the possibility of protein-caloric malnutrition, was observed significantly more frequently in the ‘high risk group”. One should remember that patients who have relatively poor nutrition are at increased risk of hypoglycemia, partly because of inadequate maintenance of muscle and liver glycogen stores [30]. Therefore it can be suspected that lower HbA_1C_ values in these cases might be the result of such.

The criteria of the “high risk group” (“age 80 + years” or “end stage renal disease” or “dementia” or “macrovascular complications”) were fulfilled by 61.03% of patients in our study. People aged 80 years or older have limited life expectancy and an increased co-morbid illness burden and thus have decreased lifetime benefit and increased risk associated with intensive glycemic control. The same concerns type 2 diabetes patients with a history of macrovascular events. Impaired renal function affects the efficacy of elimination of oral glucose lowering drugs (particularly an issue for sulfonylurea) and insulin, whereas dementia may adversely affect patients’ ability to self-manage their diabetes, and both are associated with increased risk of serious hypoglycemia [31, 32].

In the “high risk group” of patients, for which less restrictive goals of therapy were recommended, 40.8% had HbA_1C_ values ≤6.0% [42 mmol/mol], 55.4% values ≤6,5% [48 mmol/mol], and 73.1% values ≤7.0% [53 mmol/mol]. The median level of HbA_1C_ was in this group also very low- 6.4% [46 mmol/ml], interquartile range 5.7–7.3% [39-56 mmol/mol]. The “low risk group” did not differ in the percentages of increasingly tight diabetes treatment prevalence, and in median value of HbA_1C_. Taking the above into account, we may hazard a guess that the treatment of diabetes in the older people admitted to the geriatric ward (especially in very old age) was frequently too tight before hospitalization, and the HbA_1C_ levels achieved in these patients were associated with a significantly increased risk of recurrent hypoglycemia, as confirmed by studies with continuous glucose monitoring [33].

The type of glucose lowering therapy may contribute to the increased risk of hypoglycemia, and some anti-diabetic agents (metformin, alpha-glucosidase inhibitors, thiazolidinediones, GLP-1 receptor agonists, DPP-4 inhibitors) are known to be safer in this respect than others (insulin, sulfonylureas, or glinides) [34, 35]. Very frequent use of sulfonylureas (65.3% of cases), which are associated with a significantly increased risk of hypoglycemia compared to a first-line type 2 diabetes treatment drug, i.e. metformin, is noticeable in our study. This may result from a more common occurrence of coexisting diseases, renal failure in particular, in the older people, which are a contraindication to the use of biguanides (a total of 61.5% of our subjects had chronic kidney disease). However, therapy modification in accordance with the current guidelines, and inclusion of metformin in the treatment, were possible in a number of these patients at discharge (in 15.6% of cases it was started). As it turned out, 11.3% of patients required discontinuation of sulfonylureas, and in case of 7% of patients it was necessary to discontinue all glucose lowering drugs. Insulin administration has been shown to be the only significant, independent negative predictor of low HbA1c values after controlling for factors such as age, BMI, leukocyte number, low glomerular filtration rate, dementia, orthostatic hypotension, or taking sulfonylurea. This may indicate that in the case of insulin patients, self-control and supervision over therapy was better.

The strength of our study is that it included very frail older patients with a large disability burden, patients who usually are excluded from most clinical trials, and resembled the decision-making in real-life health care. It is not based on administrative claims data- the health and functional assessment performed within comprehensive geriatric assessment was multidimensional and based on different tests and scales, allowing for more reliable and in-depth analysis of health and functional correlates of diabetes therapy in the geriatric inpatients.

The study has got some limitations which have to be considered while interpreting our data. First of all, the studied group was not a random sample of the overall population of all older patients with type 2 diabetes, but only those hospitalized at a geriatric ward- in more advanced age, more disabled, and with different geriatric syndromes, such as dementia, depression, malnutrition, dependence on others in activities of daily living- ADL, and the results can be generalized for similar groups only. Although we were striving to meet the requirements of a prospective observation (i.e., all consecutive patients with diabetes admitted to our department were included), but some limitations of our study resulted from its retrospective design. The analysis of patient records did not allow, for instance, for a thorough and reliable evaluation of diabetes medications dose adjustments during hospitalization. As it was a retrospective study, it was not possible to establish who had been taking care of the patients before admission, as medical records did not include information on that.

Cross-sectional population studies of older people in Poland confirmed that they use specialist care services less frequently with the advancement of old age, and very often remain only under the care of a family doctor [36]. Such a shift of diabetic patient care is also promoted by the National Health Fund (NFZ) in Poland, which in 2008 introduced a three times higher capitation payment for family doctors who treated patients with diabetes or cardiovascular diseases. It has not been regulated, however, how these resources should be spent in the primary health care sector (i.e. how often and what type of diagnostic tests should be performed), as was emphasized by the Diabetes Poland Association [37]. In this situation, as well as in the light of the presented findings, it appears that there is an urgent need to prepare family practitioners for the appropriate management of older diabetic patients with a particular focus on the high risk groups. Such a risk group certainly includes patients of advanced old age with coexisting geriatric disabilities, also if they are not treated with insulin.

On the other hand, the above data should not be regarded as a reason for accepting higher target levels of glycated hemoglobin and less intense- or even a discontinuation of- diabetes treatment, in all geriatric patients. Uncontrolled diabetes results in metabolic disorders such as ketoacidosis, hyperosmosmolar syndromes, and dehydration, which may be life-threatening for an older person. It also significantly contributes to the development of the already mentioned geriatric comorbidities and deteriorates quality of life [12]. Therefore, an individual approach to a patient and hypoglycemic treatment modification based on the patient’s clinical condition, especially nutritional status, coexisting diseases, the degree of liver and kidney function, as well as the individual goals to be achieved are also important [38]. Better access for the older people to new hypoglycemic agents, which are associated with significantly lower risk of hypoglycemia, would also prove significant. Such medications, however, were generally not used in our patient population due to financial constraints, and treatment, as shown in our study, was limited to four groups of drugs: sulfonylurea, metformin, insulin, and – in small percentage- acarbose.

## Conclusions

In conclusion the results show a very high rate of tight glycemic control in older patients admitted to the geriatric ward, for whom higher HbA_1C_ targets are recommended according to the diabetes treatment guidelines (very advanced age, severe stage of chronic kidney disease, a diagnosis of dementia or a history of severe cardiovascular or vascular complications). This indicates the high probability of diabetes overtreatment in this group of patients, associated with a high risk of recurrent hypoglycemia and its adverse consequences. This is all the more likely because most of them received medications known to cause hypoglycemia. This points to the need of paying more attention to specific difficulties in the treatment of diabetes in older people, especially those suffering from various geriatric syndromes and diseases worsening their prognosis.

## Data Availability

The dataset(s) supporting the conclusions of this article is(are) available in the RepOD repository, [10.18150/repod.4546680].

## Abbreviations

ACCORD: Action to Control Cardiovascular Risk in Diabetes study
ADL: Activities of daily living
ADVANCE: Action in Diabetes and Vascular Disease: Preterax and Diamicron MR Controlled Evaluation study
BMI: Body mass index
CABG: Coronary artery bypass graft
CI: Confidence interval
CKD: Chronic kidney disease
DCCT: Diabetes Control and Complications Trial
DPP-4: Dipeptidyl peptidase-4
EDWPOP: European Diabetes Working Party for Older People
GDS: Geriatric Depression Scale
GFR: Glomerular filtration rate
GLP-1: Glucagon-like peptide 1
HbA1C: Glycosylated A1C hemoglobin
IQR: Interquartile range
KDOQI: Kidney Disease Outcome Quality Initiative
M: Mean
MI: Myocardial infarction
NFZ: National Health Fund in Poland
OARS: Older Americans Resources and Service
OR: Odds ratio
PTCA: Percutaneous transluminal coronary angioplasty
SD: Standard deviation
SOMCT: Short Orientation- Memory- Concentration Test
TIA: Transient ischemic attack
UKPDS: United Kingdom Prospective Diabetes Study
VADT: Veteran’s Affairs Diabetes Trial
